# Sustaining connections: feasibility and impact of long-term virtual patient engagement

**DOI:** 10.1186/s40900-024-00558-2

**Published:** 2024-02-24

**Authors:** Kelsey Stefanik-Guizlo, Claire Allen, Sarah Brush, Jessica Mogk, Starette Canada, Marina Peck, Kathryn Ramos, Karen Volpe, Paula Lozano

**Affiliations:** Kaiser Permanente Washington Health Research Institute, 1730 Minor Ave, Ste 1600, Seattle, WA 98101 USA

**Keywords:** Patient engagement, Patient and public involvement, Virtual engagement, Chronic pain

## Abstract

**Background:**

Virtual patient engagement has become more common in recent years. Emerging research suggests virtual engagement can increase accessibility for patients managing long-term health conditions and those living in larger geographic areas, but it can also be challenging to establish relationships and maintain engagement over time. Little is known about virtual engagement lasting more than two years, nor about the specific contributions of patients to virtual engagement projects. Here we describe a project where virtual engagement was sustained over a long period of time (3.5 years), measure patients’ contributions to the work, and describe the facilitators and challenges of the project using the Valuing All Voices (VAV) patient engagement framework.

**Methods:**

Five researchers recruited four patient partners living with persistent pain to work together virtually on a project to improve care for others with long-term pain. Researchers documented engagement activities and patient partner contributions and categorized them using Carman et al.’s 3 types of engagement. They also collected data via semi-structured group interviews with patient partners about the facilitators and challenges of the project using the VAV framework.

**Results:**

In 3.5 years, patient partners contributed 487 h to the project, averaging 3.0 h per month, and participated in 40 meetings. They contributed to 17 products for patients, health care teams, and researchers. Most products (12 of 17) were created using the more in-depth engagement approaches of involvement or partnership and shared leadership. The group identified facilitators of the project across the five VAV domains of relationship-building, trust, understanding & acceptance, education & communication, and self-awareness, as well as some specific challenges such as keeping track of products across virtual platforms and managing the high volume of project information.

**Conclusions:**

Long-term virtual patient engagement is feasible and can use more in-depth engagement approaches. Additionally, it can result in substantial contributions from patients in terms of time, effort, and products. These findings can inform future long-term virtual patient engagement efforts and provide insight into how researchers can structure their activities to encourage and maintain deep engagement over time.

**Supplementary Information:**

The online version contains supplementary material available at 10.1186/s40900-024-00558-2.

## Abstract (plain language summary)

**Background:**

Virtual patient engagement has become more common in recent years. Virtual engagement can make it easier for people with long-term health conditions and from larger geographic areas to be involved, but it can also be challenging to establish relationships and maintain engagement over time. There are not many examples of virtual engagement projects lasting more than two years, and many projects do not describe patients’ specific contributions to the work. Here we describe a 3.5-year project where we measured patients’ contributions and described the facilitators and challenges of the project using the Valuing All Voices (VAV) patient engagement framework.

**Methods:**

Five researchers worked with four patient partners living with persistent pain to improve health care for others with long-term pain. We tracked how we worked together and how patient partners contributed to the project. We categorized the products we created together using three types of engagement that range from less to more in-depth. Researchers also interviewed patient partners about the facilitators and challenges of working together virtually.

**Results:**

In 3.5 years, patient partners contributed 487 h to the project, averaging 3.0 h per month, and participated in 40 meetings. They contributed to 17 different products. Twelve of the products used more in-depth engagement approaches. The group identified facilitators of the project across the five VAV domains, as well as some specific challenges.

**Conclusions:**

We found that long-term virtual patient engagement is feasible, can use in-depth engagement strategies, and can result in important contributions from patients in terms of time, effort, and products.

**Supplementary Information:**

The online version contains supplementary material available at 10.1186/s40900-024-00558-2.

## Background

Patient engagement in research is defined as the active, meaningful, and collaborative interaction between patients and researchers across all stages of the project lifecycle, where decision-making is guided by patients' contributions as partners, recognizing their specific experiences, values, and expertise [1]. Patient engagement in health services research has well documented benefits for patients, researchers, and health systems [2–7]. COVID-19 changed how many researchers engage patients, with more traditional in-person interaction shifting to remote and virtual settings [8]. Literature on virtual patient engagement is still emerging [9], but documented benefits include enabling people from larger geographic areas to participate, greater accessibility of participation for people with long-term health conditions, and avoiding the time, cost, and stress of traveling to an in-person meeting [8, 10–12]. Challenges include differences in familiarity, comfort, and access to technology [8, 12], building and maintaining relationships when interactions are solely virtual [8, 9, 13–15], and sustaining active participation from patients over time [12, 16]. Little is known about virtual engagement that lasts for more than two years, possibly due to these and other challenges.

Another gap in the literature, regardless of whether the engagement was in-person or virtual, is documentation of the contributions of patients [17]. A lack of documentation makes it difficult for researchers to measure the impact of patients’ contributions and communicate it back to them and other collaborators, which may negatively impact patients’ motivation to continue working on research projects [3, 4, 14, 17, 18].

Many frameworks have been developed to characterize patient engagement activities [19]. Carman et al.’s Multidimensional Framework for Patient and Family Engagement in Health and Health Care is a well-recognized resource that was developed in collaboration with patients. The framework identifies three levels of engagement: direct care that relates to the patient’s own health care decisions and behaviors, organizational design and governance of health care organizations (clinics, hospitals, etc.), and health-related policymaking at the national, state, and local level [20]. This project was part of a health system quality improvement project and focused specifically on the organizational design and governance level of engagement. The framework also describes a continuum of engagement (consultation, involvement, and partnership and shared leadership) based on how active and involved patients are in decision making. In organizational design and governance, the lower end of the engagement continuum (consultation) may include asking patients for their input without giving them much power to influence the final decision or approach. At the higher end of the continuum (partnership and shared leadership), patients are considered co-leaders of initiatives and have shared decision-making power [20].

The Valuing All Voices (VAV) framework complements Carman’s framework by bringing a social justice and health equity perspective to engagement, outlining five categories of activities health research teams should focus on to make patient engagement more meaningful and inclusive: education and communication, understanding and acceptance, trust, self-awareness, and relationship-building [21]. These two frameworks informed the patient engagement approach for the project described here, which focused on improving care for people living with long-term pain.

The purpose of this paper is to describe how researchers and patients (who we called ‘patient partners’) from the Center for Accelerating Care Transformation at Kaiser Permanente Washington Health Research Institute (KPWHRI) worked together virtually on a long-term (3.5 year) quality improvement project to design, implement, and evaluate an integrated care program for patients with persistent pain. We present our engagement methodology in detail, along with clear documentation of patient partners’ contributions to the work categorized by Carman et al.’s continuum of engagement. Since the VAV framework was developed for in-person engagement, we also describe the facilitators and challenges of this virtual project using this framework. In our work, we use the terms ‘persistent pain’ and ‘long-term pain’ instead of ‘chronic pain’ to better represent the diverse experiences of people living with pain [22].

## Methods

### Setting and approach

This work took place within the Learning Health System program at Kaiser Permanente Washington (KPWA), an integrated health system in Washington State. [23] In response to changing federal, state, and organizational opioid guidelines and dissatisfaction from both providers and patients about the implementation of these guidelines, researchers partnered with KPWA leaders, care teams, and patients to develop and implement the Integrated Pain Management program, an evidence-based, patient-centered approach to reduce long-term opioid prescribing and improve care for patients with persistent pain. The KPWHRI Institutional Review Board determined that this quality improvement project was not human subjects research.

### Patient partner recruitment and onboarding (February–July 2020)

In forming the patient partner group, researchers sought to bring together KPWA patients with persistent pain from diverse backgrounds and medical experiences. They planned to recruit up to five patient partners who were current members of KPWA, had experience receiving care for persistent pain at KPWA, and were interested in improving pain care for patients broadly.

Starting in early 2020, researchers recruited patients through primary care providers and existing organizational groups that engaged patients. Patients who were contacted by the researchers also helped to identify additional eligible patients—one person helped recruit two people she already knew. Eleven interested patients completed interviews. Researchers extended invitations to join the project to eight candidates, four of whom (SC, MP, KR, and KV) accepted. Reasons for not accepting the invitation included health problems, burden of the onboarding process, expected time commitment of the group, and perception that program materials were stigmatizing. The four patient partners then completed a set of onboarding activities, including an orientation meeting and administrative paperwork (including a background check authorization, application form, and W-9) required for KPWA to provide compensation at a rate of $50 per hour.

### Engagement methods (June 2020–November 2023)

Researchers designed the project to focus on the organizational design and governance level of the Carman et al. framework and sought ways to move further along the engagement continuum to involvement and, where possible, partnership and shared leadership. One researcher, the patient partner coordinator (SB), was responsible for planning meetings, leading communications activities, and managing patient partner workload, with support from the other researchers as needed. Between June 2020 and April 2023, we held monthly, 60-min virtual meetings facilitated by a researcher. From April 2023 to November 2023, we held bimonthly meetings. The first 15–20 min were devoted to building interpersonal relationships through icebreaker activities and personal updates. The remainder of the meeting was used to share project updates, review patient partner feedback on project planning, activities, and products, and/or discuss news of relevance to the project, such as opioid guideline changes.

**Fig. 1:**
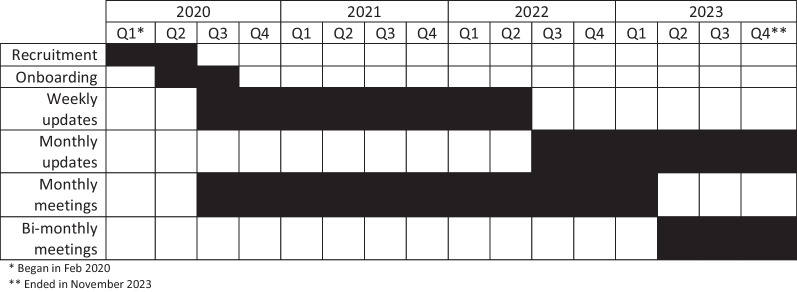
Timeline of patient engagement activities

Between meetings, the patient partner coordinator sent weekly, and later monthly, newsletters that explained key terms and policies related to pain care at KPWA, provided updates about the project, and showcased recipes and photos from group members. Over the course of the project, the coordinator sent 92 email newsletters. Patient partners were also asked to review and provide feedback on project materials. The coordinator sent documents via email and patient partners provided feedback using track changes in Microsoft Word documents, handwritten edits on printed copies, and verbal comments in meetings. Researchers compiled and applied the feedback and updated the patient partners about the resulting changes made to the materials. The patient partner coordinator also held periodic 1-on-1 check-in calls with patient partners to solicit feedback, learn about partners’ areas of interest, and provide technical support. The patient partner coordinator spent approximately 12 h per week on engagement activities during intensive times of the project (developing weekly communications, facilitating work on products, and providing ad hoc 1-on-1 support), and approximately four hours per week toward the end of the project when communications had moved to monthly and patient partners had fewer products in development. A timeline of engagement activities is detailed in Fig. 1.

In all activities, we strived for intentional power sharing, which aligns with Carman, VAV, and many other patient engagement frameworks that highlight the importance of addressing power dynamics when pursuing meaningful, in-depth engagement [8, 9, 16]. Researchers sought to de-position themselves as experts by emphasizing the unique expertise of each patient partner, encouraging them to share their perspectives and experiences, and tracking how their feedback was applied to project planning, activities, and products. During meetings, patient partners shared power by inviting each other to offer their thoughts and ideas, validating each other’s experiences, and affirming one another’s contributions.

### Data collection and analysis

A Guidance for Reporting Involvement of Patients and the Public (GRIPP 2) long form [24] is included as Additional file 1 to document how we reported our patient engagement activities in this manuscript.

### Documentation of patient partner contributions

Researchers tracked the duration of engagement, number and purpose of meetings, hours per month patient partners spent on meeting preparation, attendance, and follow-up based on invoices received from the patient partners, and the total cost of patient compensation over the project based on all invoices received. Researchers also documented the total number of project materials that were co-created or reviewed by patient partners. For each product, patient partners were asked to provide written feedback, verbal feedback during a meeting, or both. To assess the depth of patient partner engagement on the products we created, we adapted the three types of engagement on Carmen’s patient engagement continuum (consultation, involvement, or partnership and shared leadership) [20] and collaboratively categorized our products. Where possible, researchers also tracked the frequency of electronic access of the products to measure product reach.

### Patient partner interviews and analysis

Three semi-structured 90-min group interviews were led by SB to elicit patient partners’ reflections on the facilitators and challenges of the project, using VAV as a framework. As Roche et al. note, group interviews support conversation and reflection between participants and encourage “in-depth exploration and co-construction of participant perspectives, knowledge, and beliefs” [21, 25]. Patient partners also asked for opportunities to reflect together, as they found that hearing one another’s ideas and feedback was generative and often sparked additional reflections or ideas. For each VAV component, the researchers asked patient partners: (1) what did this look like for us?, (2) when did we do this well?, and (3) when did we fall short? SB captured detailed notes during the interviews, which were analyzed by KSG to identify themes. Interview notes and resulting data were reviewed for accuracy by SC, MP, KR, KV, SB, and JM.

## Results

### Patient partner and researcher characteristics

Four patient partners with lived experience with pain collaborated closely with a team of five researchers. Table 1 shows the characteristics of the group. Current ages of patient partners range from 46 to 72 (average: 64); researchers range in age from 27 to 60 (average: 39). All patient partners and researchers identified as female. Three patient partners identified as White, and one identified as Black/African American. None identified as Latine. All five researchers identified as White; one also identified as Latina. All patient partners have personal lived experience with persistent pain. No researchers have this experience, although four have family members who live with long-term pain. In addition to their lived experience with pain, each patient partner brought a range of skills to the group, including health education, plain language writing, marketing, creative writing, and public speaking. A positionality statement for each author is included in this manuscript.

**Table 1:** Characteristics of patient partners and researchers (N = 9)

	Number (range or %)	
	Patient partners	Researchers
Age		
Mean (range)	64 (46–72)	39 (27–60)
Gender		
Female	4 (100%)	5 (100%)
Male	0 (0%)	0 (0%)
Race		
Black/African American	1 (25%)	0 (0%)
White	3 (75%)	5 (100%)
Ethnicity		
Latine	0 (0%)	1 (20%)
Non-Latine	4 (100%)	4 (80%)
Lived experience with persistent pain		
Yes	4 (100%)	0 (0%)*
No	0 (0%)	5 (100%)

*No researcher had lived experience with persistent pain, but 4 of the 5 had family members who live with persistent pain

### Patient partner contributions

In 3.5 years (June 2020 through November 2023), patient partners and researchers met 40 times to discuss project updates, build relationships between one another, review patient partner feedback on project activities and products, and plan future activities. An overview of patient partner contributions is included in Table 2. The total cost of patient partner compensation over the three-year project was $24,350.

**Table 2:** Overview of patient partner engagement in the KPWA Integrated Pain Management program

Number of meetings	40
Average number of hours per month contributed by patient partners	3.0
Total number of hours contributed to project by patient partners	487 h
Duration of engagement	3.5 years (June 2020–December 2023)
Total number of co-developed products	17

Patient partners contributed to 17 products that used a combination of Carman et al.’s engagement types. Table 3 includes each product, the audience, and a brief description. Researchers engaged patient partners at varying levels of collaboration on the continuum of engagement in creating products related to the project. The highest level of engagement (partnership and shared leadership) was used for three products, involvement for nine products, and consultation for four products. Researchers and/or patient partners initiated all the products that used involvement or partnership and shared leadership approaches, while products created using consultation arose from requests by other collaborators at KPWA. While the overall patient engagement project was focused at the level of organizational design and governance, many of the resulting products are focused at the direct care level and aim to equip both patients and care teams to move along the engagement continuum so that patients are empowered and care teams have tools to treat persistent pain with a comprehensive approach. Researchers and patient partners are also collaborating on two manuscripts related to this project to be submitted to peer-reviewed journals, with the intended audience of health system leaders and researchers.

**Table 3:** Project products co-developed with patient partners, categorized by Carman’s continuum of engagement *

Product	Audience		Description	
**Partnership and shared leadership**: product was initiated by patient partners and researchers using shared power in decision-making; patient partners substantively contributed to developing the product, completed in-depth review(s), and recommended changes to ensure patient-centeredness				
Toolkit for Managing Persistent Pain	People with persistent pain		60-page toolkit with information about persistent pain, self-management strategies, treatment options, and navigating the health care system. Also includes stories, journal prompts, trackers, and checklists. Available in three versions: public, KPWA-specific, and Spanish. Accessed electronically 811 times as of November 2023	
Six-part discussion guide about bias, stigma, and racism in pain care	Primary care teams		Intended to support clinic teams in taking action to reduce bias, stigma, and racism in care for patients with persistent pain. Includes six modules of content that can be covered in 15–20 min at a huddle or clinic staff meeting	
Empathic Pain Conversations training scenario	Health care teams		Part of a two-part, two-hour training about empathic communication strategies for caring for patients with persistent pain. The scenario describes an example situation from the perspectives of a patient and a health care provider and offers recommendations from patient partners	
**Involvement**: product was initiated by researchers; patient partners completed in-depth review(s) and recommended changes to ensure patient-centeredness				
Patient education handouts [4]	People with persistent pain		Four handouts with information and resources. Topics include understanding long-term pain, complementary treatments for long-term pain, using opioid medicine for long-term pain, and educational videos for patients with long-term pain. Available in Chinese, English, Khmer, Korean, Russian, Spanish, and Vietnamese Accessed electronically 1,865 times as of November 2023	
Scripts for health care providers to help them talk with patients about sensitive topics [3]	Primary care providers		Comprehensive, patient-centered scripts to help health care providers talk to patients about their chronic opioid therapy plan, tapering opioids, and naloxone. Provides strategies for communication using the Ask-Offer-Ask approach	
Posters about the patient partners’ work for dissemination at academic conferences [2]	Health care researchers		Posters about this project were accepted and presented at the 2022 Health Care Systems Research Network conference and 2023 AcademyHealth conference	
**Consultation:** product was initiated by a person or team outside of the group; patient partners completed one-time review and offered feedback				
Journey map of persistent pain management in primary care at KPWA	Our team of researchers and patient partners		Synthesis of themes from interviews with people receiving pain management support at KPWA. Used to ground the team in the patient experience early in the project and identify opportunities for intervention	
Pain skills class marketing flyer	People with persistent pain		Recruitment flyer for a four-session virtual class led by a social worker to equip patients with self-management skills for persistent pain	
Care management for chronic pain program recruitment flyer	People with persistent pain		Recruitment flyer for a program that connects people who take daily opioid medicines for pain with a social worker and pharmacist who provide resources, support, and assistance with managing pain medicines	
Care management for chronic pain program script	Primary care providers		Script that primary care providers can use to tell patients about a program that connects people who take daily opioid medicines for pain with a social worker and pharmacist who provide resources and support, and assistance with managing pain medicines	

*Definitions for the continuum of engagement adapted from Carman et al.’s Multidimensional Framework for Patient and Family Engagement in Health and Health Care^3^

In addition to developing specific products, patient partners also contributed to the overall Integrated Pain Management program in many ways that are difficult to itemize. These include encouraging researchers to avoid stigmatizing language, holding researchers accountable for documenting and reporting back on how they applied patient partner feedback, and helping researchers consider patient perspectives when making decisions about the project and working with other partners in KPWA (e.g., clinic staff and organizational leaders).

Semi-structured group interviews revealed that patient partners valued time in meetings to collectively discuss feedback on products and hear from researchers about how their comments and edits were incorporated into drafts and final versions. During some review meetings the researchers used Miro [26], an online virtual collaboration platform, to keep track of ideas and then organize them together, which patient partners found helpful. Patient partner edits ranged from minor edits to enhance readability and increase visual appeal to larger, more comprehensive changes. For example, we changed the language in our products to use the term ‘persistent pain’ instead of ‘chronic pain’ because patient partners noted the negative connotation that usually comes with the adjective ‘chronic’ (e.g., ‘chronically late’ or ‘chronic liar’). We also shifted the voice of the Toolkit for Persistent Pain from third to first person to be more appealing to people living with pain. As a final example, based on their own experiences, patient partners saw a need for training health care teams about stigma, bias, and racism in pain care, which led to the co-production of a six-part discussion guide on these topics for primary care teams.

### Project facilitators and challenges

Patient partners identified the facilitators and challenges of this virtual engagement project, shown by VAV domain in Table 4. Facilitators included researchers engaging patients early in the process (trust), positioning patient partners as the experts (trust), and providing information about the project, its goals, the context, and the potential impact (education and communication). The group dedicated 15–20 min of each 60-min meeting to personal updates and icebreakers (relationship-building), validated and celebrated one another for showing up even when they were not feeling well (understanding and acceptance), and made sure to hear from everyone during discussions and decision-making (education and communication). Researchers and patient partners were also mindful of their positionality and privilege and spent time discussing the impact of various forms of bias and discrimination on people with pain (self-awareness). Group members embraced practicing “mess up, fess up” by admitting when they made mistakes or said something that could be hurtful to others (trust, self-awareness).

**Table 4:** Facilitators and challenges to sustaining virtual patient engagement over time, by Valuing All Voices domain

Domain	Facilitators	Challenges
Education & communication	•The group reviewed goals and consistently worked toward them •The group welcomed everyone's communication style and regularly practiced listening and asking clarifying questions •Researchers held a check-in with patient partners after the first few meetings, which set the tone for providing feedback •Researchers established bi-directional communication with patient partners by regularly asking for feedback and encouraging them to reach out with ideas, questions, and concerns	•Patient partners sometimes felt overwhelmed with the amount of project information they received •Patient partners found it challenging to locate drafts and final versions of products across electronic platforms (email and Slack) •Patient partners found it hard to track progress toward project goals •Researchers could not find a way to store all project materials in an easily accessible place
Understanding & acceptance	•The group fostered a sense of unity, practice of empathy, and a “come as you are” culture •Group members shared their unique traits and experiences, such as skills in creative writing, photography, plain-language editing, and marketing, and lived experiences with pain, which created connectedness •Patient partners could contribute to the group in a variety of ways, such as sharing feedback on co-created products via written or verbal feedback or providing specific or high-level edits •Patient partners created accountability for doing meeting pre-work—not completing pre-work impacted the meeting	•Researchers weren’t always clear about what pre-work they expected from patient partners before meetings
Trust	•Researchers engaged patients early in the project •Researchers frequently referred to patient partners as the experts •The group acknowledged each person's experiences •Patient partners demonstrated vulnerability when sharing stories that were difficult, sad, or painful •Researchers were clear about the agenda, encouraged balanced participation, respected confidentiality, took notes to track discussion and feedback, shared how input would be used, and provided updates about the impact of the group’s work	N/A
Self-awareness	•The group made space for storytelling and sharing •Patient partners asked thought-provoking questions and reflected on bias •The group gained perspectives from empathizing with others •The group regularly questioned our bias and privilege and intentionally tried to see who was not “in the room” •Group members felt personal change	•The group is not demographically diverse; there was potential for tokenizing people's experience
Relationship-building	•The group greeted each other at the start of the meetings •The group did not judge appearances •The group respected each other's perspective and experience •The group offered recognition and gratitude for the talents each person contributed •Patient partners shared homemade gifts with the group, including poetry, handmade jewelry, and stationary with nature photos	•The group tested having 15 min of pre-meeting social time to give patient partners time to connect without the researchers, but patient partners found the time to be too unstructured and didn’t feel it was needed

There were also challenges in the project (shown by VAV domain in Table 4). Patient partners sometimes felt overwhelmed by the volume of information and found it challenging to locate various drafts and final versions of products across different electronic platforms such as email and Slack (education and communication). Early in the project, the group tested having 15 min of pre-meeting virtual social time to give patient partners time to connect on video without the researchers, but patient partners found the time to be too unstructured and not necessary (relationship-building). The researchers did not always clearly convey by email what pre-work was expected before meetings (understanding and acceptance). This was not a demographically diverse group, which created a risk of tokenizing people’s experiences (self-awareness).

Patient partners identified benefits to virtual patient engagement across all five VAV domains. Virtual engagement enabled people from more geographic areas and with various obligations (work, family, etc.) to participate (understanding and acceptance, relationship-building). Patient partners could attend meetings from their own spaces where they were comfortable (self-awareness), without having to dress formally (trust). The technical features of virtual meetings, such as only one person being able to talk at a time and a group norm of using the mute function when not speaking, made it easier for all participants to be heard in a respectful way (education and communication). The virtual format was particularly beneficial on days when patient partners were experiencing increased pain—often they were still able to participate in the meetings, which wouldn’t have been possible if they needed to attend in-person (self-awareness).

Though not specific to virtual engagement, potential harm to patient partners from engagement activities emerged as an important concern. Patient partners noted it was at times difficult to be deeply engaged in a long-term, in-depth health system improvement project while simultaneously experiencing the negative impact of health system policies in their daily lives (e.g., experiencing stigma when completing required urine drug screens). Although researchers were successful in advocating for changes to address some health system harms identified by patient partners, the slow pace of change was a challenge.

## Discussion

This study found that virtual patient engagement over a long period of time is both feasible and productive. Virtual patient engagement has increased in recent years, but our project is one of few reported studies where researchers partnered closely with patients for more than two years. This may be because others have noted that a virtual setting lacks some advantages of in-person contact and creates barriers to productive engagement [8, 9, 13, 14, 18, 27, 28]. For example, in a mixed methods study of more than 200 patients and professionals, many participants reported challenges building relationships in a virtual setting, and the majority preferred a mix of virtual and in-person meetings [12]. In a 6-month community-based participatory research project that had to abruptly transition to virtual meetings due to COVID-19, the group found that engagement decreased, but dramatically improved once they went back to meeting in person [16]. In contrast, we were able to maintain active engagement throughout our 3.5-year project.

Our success in maintaining virtual engagement over time may be in part due to our use of two helpful frameworks: Carman et al.’s Multidimensional Framework for Patient and Family Engagement in Health and Health Care and VAV [20, 21]. Both frameworks were developed in partnership with patients and strive to support researchers in partnering with patients in a thoughtful, intentional way, which previous research has shown is critically important [19, 29]. While neither was developed specifically for virtual engagement, we found both frameworks to be valuable in planning our engagement approach. The Carman et al. framework helped researchers think about ways to advance along the continuum of engagement to more deeply engage patient partners in organizational design and governance, and we also co-produced products to help patients and care teams to move further along the continuum at the level of direct care. The five VAV domains of trust, self-awareness, empathy, relationship-building, and education and communication provided specific areas for researchers to focus on when planning virtual patient engagement activities and helped build group cohesion and trust, despite not having face-to-face interaction. Consistent with previously published literature on virtual engagement [8, 10–12], we also identified benefits specific to virtual engagement such as allowing people from larger geographic areas and with varying work and family obligations to participate in the project and enabling people who live with persistent pain to join meetings even on days when they were having increased pain, which would not have been possible if meetings were in-person.

Documentation of patients’ contributions to projects is a commonly reported challenge in the literature [22] regardless of whether engagement was in-person or virtual. Our project aimed to document and report patients’ contributions on multiple levels to help fill this gap. In addition to tracking quantitative data such as the number of hours per month our patient partners spent on the project and the total number of meetings, we also co-created 17 products and characterized how we developed these using Carman’s engagement types. We primarily employed more in-depth types of engagement, either involvement or partnership and shared leadership, for the products we created. In alignment with best practices identified by numerous other researchers [3, 14, 18], researchers in this project reported back to patient partners about how their feedback was applied, which built trust, demonstrated that patient partners were true collaborators, and helped to ensure that our final products aligned with patient partner perspectives. The resulting products benefitted from the depth of patient partners’ involvement—they included accessible, non-stigmatizing language and presented information in a patient-centered way. The fact that products were co-produced with patient partners also generated interest among care teams and patient groups at KPWA and the broader community, and researchers in turn reported this impact back to patient partners.

This work has several limitations. First, the group is small and not demographically diverse, particularly in terms of race, ethnicity, age, and gender. As a result, there is a potential for tokenizing people’s experiences and/or not being able to bring the perspectives of others into the conversations. While we actively sought to mitigate this limitation by considering the perspectives of those who were not represented, this approach cannot substitute for diverse representation. Second, one patient partner helped recruit two people she already knew, which, as Jones and colleagues similarly reported in their work [12], may have made it easier for those group members to build trust and start engaging deeply in the work. Replicating this project in other settings might require more time to allow patient partners to build relationships with one another.

## Conclusions

We demonstrated that long-term virtual patient engagement is feasible and can use more in-depth engagement approaches. Additionally, we documented the contributions of patient partners in terms of time, effort, and products to demonstrate the substantial value they added to the project. Our findings can inform future long-term virtual patient engagement efforts and provide insight into how researchers can structure their activities to encourage and maintain deep engagement over time.

### Supplementary Information

**Additional file 1.** GRIPP2 long form.

## Data Availability

Data sharing is not applicable to this article as no datasets were generated or analyzed during the current study.

## Abbreviations

VAV: Valuing All Voices
KPWHRI: Kaiser Permanente Washington Health Research Institute
KPWA: Kaiser Permanente Washington
GRIPP 2: Guidance for Reporting Involvement of Patients and the Public
